# Protein-protein interaction network analysis of cirrhosis liver disease

**Published:** 2016

**Authors:** Akram Safaei, Mostafa Rezaei Tavirani, Afsaneh Arefi Oskouei, Mona Zamanian Azodi, Seyed Reza Mohebbi, Abdol Rahim Nikzamir

**Affiliations:** 1 *Faculty of Paramedical Sciences, Shahid Beheshti University of Medical Sciences, Tehran, Iran*; 2 *Proteomic Research Center, Shahid Beheshti University of Medical Sciences, Tehran, Iran*; 3 *Basic and Molecular Epidemiology of Gastrointestinal Disorders Research Center, Research Institute for Gastroenterology and Liver Diseases, Shahid Beheshti University of Medical Sciences, Tehran, Iran*; 4 *Faculty of Medicine, Shahid Beheshti University of Medical Sciences, Tehran, Iran*

**Keywords:** Cirrhosis, Gene ontology, Protein-protein interaction network, DAVID Bioinformatics Resources 6.7

## Abstract

**Aim:**

Evaluation of biological characteristics of 13 identified proteins of patients with cirrhotic liver disease is the main aim of this research.

**Background:**

In clinical usage, liver biopsy remains the gold standard for diagnosis of hepatic fibrosis. Evaluation and confirmation of liver fibrosis stages and severity of chronic diseases require a precise and noninvasive biomarkers. Since the early detection of cirrhosis is a clinical problem, achieving a sensitive, specific and predictive novel method based on biomarkers is an important task.

**Methods:**

Essential analysis, such as gene ontology (GO) enrichment and protein-protein interactions (PPI) was undergone EXPASy, STRING Database and DAVID Bioinformatics Resources query.

**Results:**

Based on GO analysis, most of proteins are located in the endoplasmic reticulum lumen, intracellular organelle lumen, membrane-enclosed lumen, and extracellular region. The relevant molecular functions are actin binding, metal ion binding, cation binding and ion binding. Cell adhesion, biological adhesion, cellular amino acid derivative, metabolic process and homeostatic process are the related processes. Protein-protein interaction network analysis introduced five proteins (fibroblast growth factor receptor 4, tropomyosin 4, tropomyosin 2 (beta), lectin, Lectin galactoside-binding soluble 3 binding protein and apolipoprotein A-I) as hub and bottleneck proteins.

**Conclusion:**

Our result indicates that regulation of lipid metabolism and cell survival are important biological processes involved in cirrhosis disease. More investigation of above mentioned proteins will provide a better understanding of cirrhosis disease.

## Introduction

 Cirrhosis is the advanced stage of liver fibrosis. In fibrosis, damaged tissues are replaced by collagen layers and lead to deficiency of the liver cell function. Decompensated cirrhosis may lead to hepatocellular carcinoma (HCC) (1). Since HCC is the most common intra-abdominal malignancy in the word and mortality range of liver cancer based on cirrhosis is developing, so designing and focusing on molecular research in liver disease such as cirrhosis is critical (2, 3). Liver Parenchymal cells are damaged by inflammatory reactions that can induce collagen synthesize, as well as a broad range of inflammatory cytokines and chemokines secretion (4). In Cirrhosis, normal liver architecture is disrupted by both fibrotic bands and disorganized nodules. Currently, no medical treatment for rebounding of cirrhotic changes is available (5). Cirrhosis usually occurs as a complication of previous chronic liver disease, such as autoimmune hepatitis, non-alcoholic fatty liver disease, hepatitis B or C viral infections (6). Now, the diagnostic information for cirrhosis is based on combined results of clinical test and imaging (1, 7). However, due to some limitations, these methods cannot be satisfactorily applied to a sensitive clinical diagnosis (8, 9). The liver biopsy is a diagnostic gold standard for determining liver disease severity, but this method is an invasive approach. Efforts have been focused on finding sensitive and specific predictive markers for early and non-invasive diagnosis of hepatic diseases (10). For this purpose, recognition of cirrhotic molecular pathways and their relations can be helpful to understand pathophysiological liver disease, early stage diagnosis and treatment in time. In recent years, related genes with cirrhosis have been introduced, including: apolipoprotein C-III, calponin 1, microfibrillar-associated protein 4, complement complement 7, lectin, galactoside-binding, soluble, 3 binding protein , lectin, galactoside-binding soluble 4 (Galectin-4), prolyl 4-hydroxylase, alpha polypeptide I, apolipoprotein A-I, apolipoprotein A-IV, transgelin, tropomyosin 2 and tropomyosin 4 (5, 11-15). Introducing a biomarker panel for some diseases is an important goal in diagnostic and therapeutic aspects of medicine (16). Bioinformatics is one of the novel tools in research of the modern world that analyze high throughput data in a short time (17). Enrichment analysis of interest proteins can be helpful in understanding the significant intricate parts of cells and the underlying mechanism of the disease pathology. Many investigations on disease-related genes have been performed using enrichment analysis methods. According to these investigations, there are common relations and associations between an experimentally protein/gene set of interest and a database of gene/protein sets. (18, 19). In this study, the enrichment analysis of identified proteins based on the GO and PPI are investigated to introduce some related molecular biomarkers (as a panel) to cirrhosis. 

## Materials and Methods

Using Google Scholar and PubMed are selected as search engians for protein identification. These proteins are expressed differentially in cirrhosis patients relative to the controls. 

 Analysis were performed using: STRING 9.1 (http://string-db.org/), Uniprot protein database (www.uniprot.org), EXPASY and DAVID Bioinformatics Resources (v 6.7) (http://david.abcc.ncifcrf.gov.). 

Names of related proteins were searched in uniprot and codes were extracted. The codes used in DAVID Bioinformatics Resources for GO analysis. A pack of gene annotations (e.g. functions, processes) can help identify interesting features. However, the prominent features are required for aqurate interpretation. Thus, a method is required for routine analysis of such datasets. Gene Ontology (GO) as a common vocabulary for annotation allows to identify semantically related genes and gene products (20). There are separate hierarchies for Molecular Functions (MF), Cellular Components (CC)  and Biological Processes (BP) (21). In fact, the DAVID Gene Functional Classification Tool (http://david.abcc.ncifcrf.gov) provides a list of associated biological terms into organized classes of related genes using a novel algorithm (22). Functional annotation software DAVID online program can provide functional information as clusters of sets of biological terms with similar meaning (23-25). Protein–protein associations can provide a clear point by grouping and organizing all protein-coding genes in a genome that can be assembled into a large network (26). The STRING database is designed to assemble and evaluate protein–protein association information (27). STRING 9.1 was used for illustration of predicted interactions of identified proteins and neighbor genes (28, 29). The PPI network was visualized using the Cytoscape 3.2.1 software. MINT, Reactome-Fls, databases were used for this topology visualization. 

## Results

Selected reported cirrhosis proteins (the proteins with significant effect) and their Uniprot IDs are tabulated in table 1. 

**Table 1:** The selected cirrhosis proteins and their Uniprot IDs

Geneotype	Uniprot ID	References
apolipoprotein A-I	P02647	(5)
apolipoprotein A-IV	P06727	(5)
apolipoprotein C-III	P33622	(11)
calponin 1	P51911	(12)
complement 7	P10643	(11)
fibroblast growth factor receptor 4	P22455	(30)
lectin, galactoside-binding, soluble, 3 binding protein(Galectin-3-binding protein)	Q08380	(31)
lectin, galactoside-binding, soluble, 4 (Galectin-4)	P56470	(11)
microfibrillar-associated protein 4	P55083	(12)
prolyl 4-hydroxylase, alpha polypeptide I	P13674	(12)
Transgelin	Q01995	(12)
tropomyosin 2 (beta)	P07951	(12)
tropomyosin 4	P67936	(12)

**Table 2:** The selected cirrhosis proteins and their correspond gene ontology information

	Uniprot ID: P02647
Molecular Function	Steroid binding, sterol binding, lipoprotein(receptor) binding, lipid binding, cholesterol binging,(lipid, sterol, cholesterol ) transporter activity
Cellular Component	Extracellular region, endoplasmic reticulum(lumen),plasma membrane, organelle lumen, extracellular region part, intera cellular organelle lumen
Biological Process	Regulation of protein amino acid phosphorylation, immune response , cholesterol , steroid and lipid, metabolic process , cell motion, G-protein coupled receptor protein signaling pathway, regulation of hormone levels, very-low-density lipoprotein remodeling,, cellular amino derivative metabolic process, cholesterol homeostasis, positive regulation of catalytic activity, regulation of system process, cell motility, chemical homeostasis, regulation of cytokine secretion , protein stabilization, negative regulation of cellular component organization, trans membrane transport, lipid homeostasis, sterol homeostasis, regulation of cellular localization, macromolecular complex assembly
	Uniprot ID: 06727
Molecular Function	Transporter activity for lipid, sterol and cholesterol, lipid binding, ion binding, cation binding, amine binding, alcohol binding, metal ion binding
Cellular Component	extracellular region, extracellular space, endoplasmic reticulum lumen, membrane-enclosed lumen, protein-lipid complex, plasma lipoprotein particle, very-low-density lipoprotein particle, high-density lipoprotein particle, chylomicron, , extracellular region part, intracellular organelle lumen
Biological Process	Cellular response to oxidative stress, cellular amino derivative metabolic process, regulation of system process, metabolic process, immune response, cell adhesion, leukocyte adhesion, cell – cell adhesion, regulation of cholesterol absorption, regulation of lipid catabolic process, regulation of molecular function and assembly subunits, regulation off fatty acid biosynthetic process, chemical hemostasis, catabolic process, lipid hemostasis, sterol hemostasis
	Uniprot ID: P33622
Molecular Function	Llipid binding
Cellular Component	extracellular region, extracellular space, protein-lipid complex, plasma lipoprotein particle, very-low-density lipoprotein particle, triglyceride-rich lipoprotein particle, chylomicron
Biological Process	lipoprotein trygriceride mobilization, lipid transport, lipid localization, catabolic process of lipid , glycerol , acyl glycerol and triglyceride
	Uniprot ID: P51911
Molecular Function	Actin binding, calmodulin binding, cytoskeleton protein binding
Cellular Component	Cytoskeleton
Biological Process	cytoskeleton organization, actin filament-based process, actin cytoskeleton organization, actomyosin structure organization, regulation of system process
	Uniprot ID: P10643
Molecular Function	Actin binding, calmodulin binding, cytoskeleton protein binding
Cellular Component	Extra cellular region, membrane attack complex, plasma membrane
Biological Process	lymphocyte mediated immunity, acute inflammatory response , proteolysis, cellular ion homeostasis complement activation, ,cell death, B cell mediated immunity, cellular homeostasis, cytolysis, cellular , homeostatic process1, chemical homeostasis, ion homeostasis, protein maturation, metal ion homeostasis, sodium ion homeostasis, cellular chemical homeostasis
	Uniprot ID: P22455
Molecular Function	Nucleotide binding, ATP binding, fibroblast growth factor binding, growth factor binding, protein kinase activity
Cellular Component	plasma membrane, integral to plasma membrane, integral to membrane, intrinsic to membrane, intrinsic to plasma membrane, plasma membrane part
Biological Process	Cell fate specification, cell surface receptor linked signal transduction, cell- cell signaling, phosphorylation, developmental induction, cell proliferation, respiratory system development
	Uniprot ID: Q08380
Molecular Function	scavenger receptor activity
Cellular Component	extracellular region, proteinaceous extracellular matrix, extracellular space, extracellular matrix, extracellular region part
Biological Process	defense response, cell adhesion, biological adhesion
	Uniprot ID: P56470
Molecular Function	sugar binding
Cellular Component	cytosol, plasma membrane
Biological Process	cell adhesion, biological adhesion
	Uniprot ID: P55083
Molecular Function	Fibrinogen, alpha/beta/gamma chain, C-terminal globular, Fibrinogen, alpha/beta/gamma chain, C-terminal globular, subdomain 1
Cellular Component	micro fibril, extracellular region, extracellular matrix, fibril, extracellular matrix part
Biological Process	cell adhesion, biological adhesion
	Uniprot ID: P13674
Molecular Function	cellular amino derivative metabolic process, iron ion binding, oxidoreductase activity, vitamin binding, peptidyl-prolin 4 dioxygenase activity, ion binding, cation binding, metal ion binding, transition metal ion binding
Cellular Component	mitochondrion, endoplasmic reticulum, endoplasmic reticulum lumen, membrane-enclosed lumen, organelle lumen, endoplasmic reticulum part, intracellular organelle lumen,
Biological Process	cellular amino derivative metabolic process , peptidyl-proline modification , collagen organization,oxidation reduction, extracellular structure and matrix organization
	Uniprot ID: Q01995
Molecular Function	actin binding, cytoskeletal protein binding
Biological Process	muscle organ development
	Uniprot ID: P07951
Molecular Function	structural molecular activity, actin binding, cytoskeletal protein binding, structural constituent of muscle
Cellular Component	cytoskeleton, muscle thin filament tropomyosin, striated muscle thin filament, actin cytoskeleton, myofibril, sarcomere, (intracellular)non-membrane-bounded organelle
Biological Process	regulation of hydrolase activity, regulation of ATP activity
	Uniprot ID: P67936
Molecular Function	actin binding, cytoskeletal protein binding, structural molecular activity calcium ion binding, structural constituent of muscle , ion binding, cation binding, metal ion binding
Cellular Component	cytoskeleton, muscle thin filament tropomyosin, striated muscle thin filament, actin cytoskeleton, myofibril, sarcomere, cytoskeletal part, contractile fiber part
Biological Process	cell motion

**Table 3:** Highly integrated enrichment clustering based on GO annotation for the selected proteins by the use of DAVID program. Molecular Functions (MF), Cellular Components (CC) and Biological Processes (BP) show in each cluster separately

Benjamini	P-value	Enrichment score:1.72	Annotation cluster 1
9.0E-1	1.7E-2	Cell adhesion	GOTEARM_BP_FAT
7.9E-1	1.7E-2	Biological adhesion	GOTEARM_BP_FAT
3.3E-1	2.5E-2	Extracellular region part	GOTEARM_CC_FAT
Benjamini	P-value	Enrichment score:1.37	Annotation cluster 2
3.3E-1	2.5E-2	Extracellular region part	GOTEARM_CC_FAT
3.8-E1	4.0E-2	Extracellular region	GOTEARM_CC_FAT
5.5E-1	8.0E-2	Extracellular space	GOTEARM_CC_FAT
Benjamini	P-value	Enrichment score:1.25	Annotation cluster 3
6.3E-2	1.4E-3	Endoplasmic reticulum lumen	GOTEARM_CC_FAT
8.8E-1	7.7E-3	cellular amino acid derivative metabolic process	GOTEARM_BP_FAT
4.3E-1	2.3E-2	Endoplasmic reticulum part	GOTEARM_CC_FAT
7.1E-1	1.4E-1	Endoplasmic reticulum	GOTEARM_CC_FAT
9.5E-1	3.6E-1	Intracellular organelle lumen	GOTEARM_CC_FAT
9.4E-1	3.7E-1	organelle lumen	GOTEARM_CC_FAT
9.2E-1	3.8E-1	Membrane-enclosed lumen	GOTEARM_CC_FAT
Benjamini	P-value	Enrichment score:1.72	Annotation cluster 4
3.8E-1	4.0E-2	Extracellular region	GOTEARM_CC_FAT
9.5E-1	6.3E-2	Chemical homeostasis	GOTEARM_BP_FAT
9.9E-1	1.2E-1	Homeostatic process	GOTEARM_BP_FAT
Benjamini	P-value	Enrichment score:0.17	Annotation cluster 5
1.0E0	6.7E-1	Metal ion binding	GOTEARM_MF_FAT
1.0E0	6.7E-1	Cation binding	GOTEARM_MF_FAT
1.0E0	6.9E-1	ion binding	GOTEARM_MF_FAT

The provided Gene ontology (GO) information, including biological processes (BP), cellular components (CC), and molecular function (MF) of proteins are identified and illustrated in table 2. The studied proteins based on GO annotation were divided into five clusters using of the DAVID program (see table 3). 

**Figure 1:**
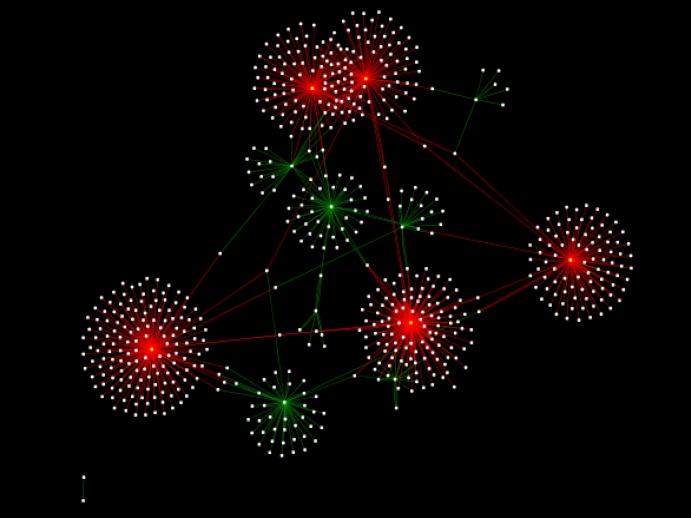
PPI network for cirrhosis obtained from MINT, Reactome-Fls and STRING databases by the application of Proteomics Standard Initiative Common QUery InterfaCe (PSICQUIC) source for the selected proteins. The network consists of 642 nodes and 926 edges. Cytoscape 3.2.1 software was used. The red points are hub-bottleneck proteins (they are listed in table 4

**Figure 2:**
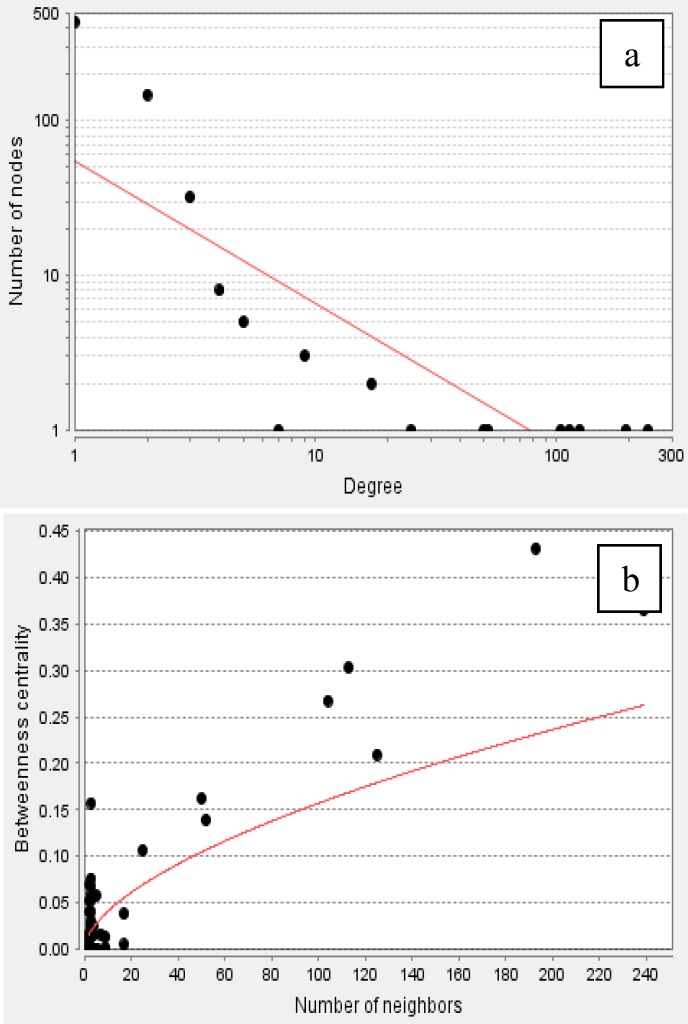
The distribution implies on the presents of proteins with high centrality values computed by Network nalyzer. The red line indicates the power law. In figure (a) the degree distribution in the scalefree network is significantly inhomogeneous. The Rsquared value is computed on logarithmized values which is equal to 0.684 and the correlation= 0.925

**Figure 3:**
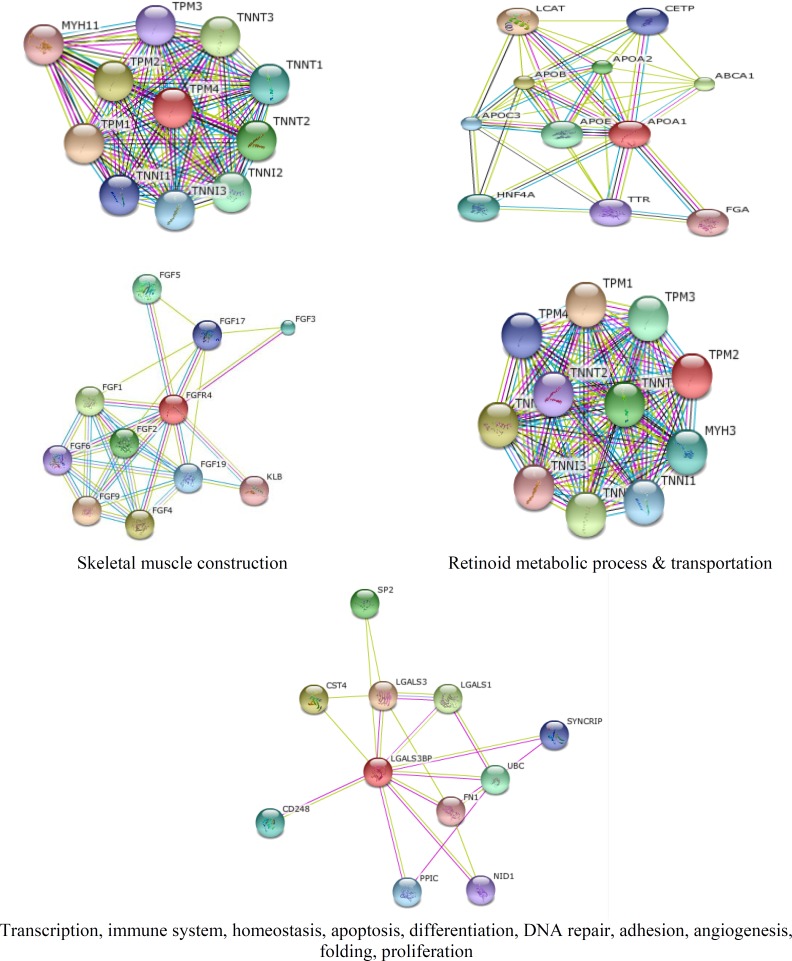
Predicted interactions for hub-bottleneck proteins (the red colored ones) with their neighboring ones were obtained from STRING online database (http://string-db.org). The related pathways of hub neighbors were obtained from QUICK GO and represented in boxes

The integrated protein-protein interaction network was obtained from MINT, Reactome-Fls, and STRING databases using Proteomics Standard Initiative Common QUery InterfaCe (PSICQUIC) source (figures 1-3). Based on centrality parameters of the network (Degree and Betweeness), fibroblast growth factor receptor 4 (FGFR4), tropomyosin 4 (TPM4), tropomyosin 2 (beta) (TPM2), *Lectin galactoside*-*binding soluble 3 binding protein* (LGALS3BP) and apolipoprotein A-I (APOA1) are identified as hubs and bottlenecks (and also as hub-bottleneck elements) of network (table 4). Evaluation of protein- protein interactions provides excellent information about its role in the systematic function of protein network. STRING resource is a suitable toll for showing these interactions (10). Using STRING, the possible interactions for hub-bottleneck proteins are presented in figure 3. 

## Discussion

Hepatic cirrhosis is a life-threatening disease arising from different chronic liver disorders. Liver cancer might occur as an end stage of steatosis, inflammation, fibrosis, and cirrhosis disease (32). Only a liver biopsy provides a reliable evaluation in grading inflammation and staging fibrosis. Therefore, non-invasive serum biomarkers for hepatic fibrosis with high sensitivity and specificity are needed(12). The use of annotation methods (mapping genes /proteins by gene ontology [GO]) can be helpful in understanding and gaining a better view of biological features of the interest sets of proteins (33).

Various factors such as oxidative stress, altered nuclear receptors, cytokines signaling, mitochondrial / peroxisomal abnormality, hepatocyte apoptosis, and leptin resistance are responsible for progression towards inflammation and fibrosis/cirrhosis (34-38). Peroxisome proliferator-activated receptors (PPARs) regulate a whole spectrum of physiological functions, including: lipid and glucose metabolism, cholesterol and bile acid homeostasis, regenerative mechanisms, cell differentiation, and inflammatory responses specifically in the liver (39, 40). Dysregulations of the expression, or activity of specific PPAR isoforms are also accepted to represent critical mechanisms contributing to the development of a wide range of liver diseases (41). As it is listed in table 1, there are 13 proteins- related to cirrhosis disease. However, additional investigation will be needed to elevate this number. According to DAVID information (see table 2), apolipoprotein A-I and apolipoprotein C-II appropriate in the PPAR signaling pathway; therefore, their related proteins may play a critical role in liver diseases. Previous studies showed that elevation of apolipoprotein A-I concentration is related to the degree of liver injury  (25) . FGFR4, ubiquitous protein that has a key role in extracellular matrix (ECM) turnover during fibrogenesis, may be associated with the risk of HCC coupled with liver cirrhosis (42, 43) and cirrhosis (30). 

FGFR4 contributes in the MAPK signaling pathway (25). Further understanding of common pathways in related proteins with special disease is essential for application in clinical settings. Recent studies have indicated MAPK signaling pathways play key roles and act as therapeutic targets in liver injury (44).  As findings indicate, these studied proteins belong to PPAR signaling, MAPK signaling, Endocytosis, regulation of actin cytoskeleton, arginine and proline metabolism, drug metabolism, cardiac muscle contraction and hypertrophic cardiomyopathy (HCM) pathway. 

Proteins with high degree are in the right down region of the plot. In figure (b) the betweenness centrality (network nodes that have many “shortest paths”) that can be considered in the range of 0-1, show the distribution 0.0 - 0.431. The R-squared value is computed on logarithmized values which is equal to 0.338 and the correlation= 0.928. Proteins with high betweenness are in the right-up region of the plot.

According to DAVID, Based on GO analysis, most of the proteins are located in the endoplasmic reticulum lumen, intracellular organelle lumen, membrane-enclosed lumen and extracellular region. Molecular function analysis showed that actin binding, metal ion binding, cation binding, ion binding are the involved function in this liver disease. The relevant biological processes are cell adhesion, biological adhesion, cellular amino acid derivative metabolic process, chemical homeostasis and homeostatic process. Adhesion molecules are glycoproteins in the surface of cells that are essential for the leukocytes localization at sites of inflammation (45).  In polycystic liver disease, the overexpression of growth factor receptors and loss of adhesion were reported (46). Alterations in inflammation-related components and soluble adhesion molecules are prognostic significance in the cirrhosis disease. Systemic inflammation is one of the significant elements that are involved in cirrhosis physiopathology. Systemic inflammation plays a considerable role in the cirrhosis-associated immune dysfunction syndrome (47). Some of the studied proteins are Figure 2**.** The distribution implies on the presents of proteins with high centrality values computed by Network Analyzer. The red line indicates the power law. In figure (a) the degree distribution in the scale-free network is significantly inhomogeneous. The R-squared value is computed on logarithmized values which is equal to 0.684 and the correlation= 0.925. Proteins with high degree are in the right down region of the plot. In figure (b) the betweenness centrality (network nodes that have many “shortest paths”) that can be considered in the range of 0-1, show the distribution 0.0 - 0.431. The R-squared value is computed on logarithmized values which is equal to 0.338 and the correlation= 0.928. Proteins with high betweenness are in the right-up region of the plot. involved in the inflammatory response, while others are involved in lipid transport activity. They can effect on lipid composition of cellular membranes. This process changes plasma lipid and lipoproteins level (48).  As it is depicted in tables 2 and 3, cirrhosis disease is characterized by the vast alterations in molecular functions, cellular components and biological processes. PPI network for cirrhosis disease (see figure 1) introduced 642 nodes and 926 edges. Topological analysis leads to determination of five hub-bottleneck proteins. These key proteins are tabulated in table 4. A hub protein is a node with a number of links that greatly exceeds the average (49). APOA1 as a hub protein possess highest degree value (degree is one of the centrality parameters). ApoA1 is the main protein component of high density lipoprotein in plasma (50), which is involved in the formation of most plasma cholesterol esters (51). A bottleneck protein plays a critical role in the integrity of the network. Lectin, galactoside-binding soluble 3 binding protein (LGALS3BP) is characterized by highest betweenness value (betweenness is the other parameter of centrality properties of network). LGALS3BP is involved in defense response, cell adhesion and biological adhesion processes. Possible interactions with neighboring proteins for hub-bottleneck proteins (see figure 3) provided valuable information for evaluation of the biological importance of these proteins. According to STRING database information (figure 3), related proteins of 5 hub- bottleneck have been predicted. Pathways of hub neighbors were obtained from the QUICK GO (a web-based tool that allows easy browsing of the Gene Ontology) (52) proteins involved in the same pathway except LGALS3BP (figure 3). Related proteins with TPM4 and FGFR4 involved in skeletal muscle contraction and MAPK cascade, respectively. For APOA1, related proteins belong to retinoid metabolic process and transportation. The results of related proteins in TPM2 are the same TPM4. Neighbors of hub proteins participate in the same pathway and the same function. Controlling the expression of these five proteins has considerable effects on pathology of cirrhosis disease. This achievement requires more investigation, especially following patients in the process of disease development. 

**Table 4:** Hub-bottleneck proteins with significant centrality values, based on two fundamental centrality properties Degree and Betweenness

Protein name	Degree	Betweenness
FGFR4	104	0.267
TPM4	113	0.303
TPM2	125	0.209
LGALS3BP	193	0.431
APOA1	239	0.365

In the clinical usage, liver biopsy (invasive method) still remains the gold standard for diagnosis of hepatic fibrosis. Biomarker discovery and molecular investigation are powerful tools in diagnosis and treatment of this disease. Protein-protein interaction network analysis can elevate understanding of molecular events. Here, five proteins relative to cirrhosis were introduced as hub-bottleneck protein. It can be concluded that regulation of gene expression, including FGFR4, TPM4, TPM2, LGALS3BP and APOA1 proteins can play a key role in the pathology of cirrhosis disease. These findings indicated that the studied proteins belong to PPAR signaling, MAPK signaling, Endocytosis, regulation of actin cytoskeleton, arginine and proline metabolism, drug metabolism, muscle contraction and hypertrophic cardiomyopathy (HCM) pathway.

## Note

**(**Please cite as:** Safaei A, Rezaei Tavirani M, Arefi Oskouei A, Zamanian Azodi M, Mohebbi SR, Nikzamir AR. Protein-protein interaction network analysis of cirrhosis liver disease. Gastroenterol Hepatol ****Bed Bench**** 2016;9(2):114-123).**
